# Neuromuscular Responses to Unilateral and Bilateral Execution of Eccentric Exercises: A Multidimensional sEMG Study

**DOI:** 10.3390/sports13100364

**Published:** 2025-10-15

**Authors:** Yanan You, Dai Sugimoto, Norikazu Hirose

**Affiliations:** 1 Graduate School of Sport Sciences, Waseda University, Tokorozawa 359-1192, Saitama, Japan; yanan.you@ruri.waseda.jp; 2 Division of Sports Medicine, Boston Children’s Hospital, Boston, MA 02115, USA; dai.sugimoto.007@gmail.com; 3 The Micheli Centre for Sports Injury Prevention, Waltham, MA 02453, USA; 4 Faculty of Sport Sciences, Waseda University, Tokorozawa 359-1192, Saitama, Japan

**Keywords:** electromyography, exercise therapy, hamstring muscles, muscle contraction, resistance training, sports medicine

## Abstract

Hamstring injuries are frequent in sports, often linked to eccentric overloading during sprinting. While eccentric strengthening, like Nordic curls and hip extensions, is common, the impact of exercise symmetry (unilateral vs. bilateral) on neuromuscular control remains unclear. This study aimed to investigate regional/task-specific neuromuscular strategies during unilateral and bilateral eccentric loading of the same exercises. Twenty-five healthy and physically active young men (age: 24.52 ± 3.82 years; height: 175.53 ± 5.44 cm; weight: 72.06 ± 7.44 kg) were recruited based on physical activity screening, with the exclusion criteria including recent lower limb injuries. Participants performed unilateral and bilateral curls and extensions with surface electromyography on hamstrings, gluteus maximus, and trunk stabilisers. Parameters like root mean square and median frequency were extracted and statistically compared. Unilateral execution generally elicited higher muscle activation, particularly in middle hamstring regions (30.65% to 38.38% in RMS, *r* = −0.84 to −0.77, *p_FDR_* < 0.001). Frequency differences revealed region-specific neuromuscular strategies. Intra-hamstring comparisons revealed significantly higher median frequencies in the BF50 and ST30 regions at their respective anatomical locations (*dz* = −1.90 to 1.34, all *p_FDR_* < 0.001). These findings suggest that exercise symmetry and anatomical specialisation jointly shape neuromuscular control, with implications for designing eccentric training to reduce injury risk.

## 1. Introduction

Hamstring injuries represent one of the most common musculoskeletal problems in sports, accounting for a high proportion of time-loss injuries and demonstrating recurrence rates as high as 20% to 30% [1,2]. These injuries are particularly frequent during high-speed running and kicking, where the hamstrings must withstand large eccentric forces [3,4]. Despite the implementation of preventive strategies, hamstring injuries remain a persistent challenge for athletes and clinicians [1,5,6]. This highlights the need for a deeper understanding of how different training modalities influence neuromuscular control and the development of more targeted eccentric exercise prescriptions.

Eccentric strengthening of the hamstrings is widely recognised as a cornerstone for both performance optimisation and injury prevention [7,8]. Anatomical evidence shows that the hamstrings are regionally heterogeneous, with proximal regions contributing more to hip extension and distal regions being more active during knee flexion [9]. Such functional differentiation may explain why the distal musculotendinous junction is particularly vulnerable to strain injuries [10,11]. Region-specific eccentric training approaches have therefore attracted growing attention for their potential to address site-specific injury risk and to enhance rehabilitation outcomes [10,12,13].

The Nordic hamstring exercise (NHE) and eccentric hip extension (EHE) are two well-established eccentric protocols shown to improve hamstring strength and reduce injury incidence [14,15]. However, the majority of research has investigated bilateral execution, which may not reflect the unilateral loading demands of sporting movements, such as sprinting or kicking [16]. To address this limitation, unilateral variants of NHE and EHE have been introduced. Yet, how movement symmetry (unilateral vs. bilateral) modulates regional activation when the exercise type itself is held constant remains understudied. This gap restricts the translation of research findings into sport-specific training and rehabilitation programs [17,18,19].

Surface electromyography (sEMG) provides a non-invasive means to quantify amplitude-, timing-, and frequency-based aspects of muscle activation, allowing for the assessment of neuromuscular strategies across regions and loading conditions [20,21]. Using sEMG across mid and distal hamstring sites, this study aimed to clarify how unilateral and bilateral eccentric loading affect regional muscle activation. Specifically, the following was asked:

1. Does unilateral eccentric loading lead to greater regional activation than bilateral loading within the same exercise?
2. Do sEMG-derived parameters reflect distinct neuromuscular strategies across different modes?

We hypothesised that unilateral execution would elicit higher activation and earlier recruitment, particularly in distal hamstring regions, compared with bilateral execution [17,18,19,20,21]. By clarifying the effects of movement symmetry within identical exercise types, this study contributes novel, clinically relevant insights into eccentric training design for performance enhancement, injury prevention, and rehabilitation.

## 2. Materials and Methods

### 2.1. Study Design

This study adheres to the EQUATOR Network’s reporting guidelines. As the design was a controlled laboratory-based experimental study with a randomised sequence of tasks, the CONSORT 2025 Statement for controlled trials (e.g., crossover trials) was followed to guide transparent reporting of the methodology, randomisation, and statistical analyses. The completed CONSORT checklist has been included in the Supplementary Materials. The study was not prospectively registered in a clinical trial database (e.g., ClinicalTrials.gov) because it did not involve clinical interventions or patient outcomes.

Rather than comparing the two exercises directly, this study focused on unilateral vs. bilateral execution within each exercise to examine the effect of movement symmetry on regional hamstring activation. The inclined board in the Inclined NHE (INHE) enabled safe unilateral performance while preserving eccentric loading, allowing within-exercise comparisons without inter-exercise confounds.

### 2.2. Participants

**Figure 1:**
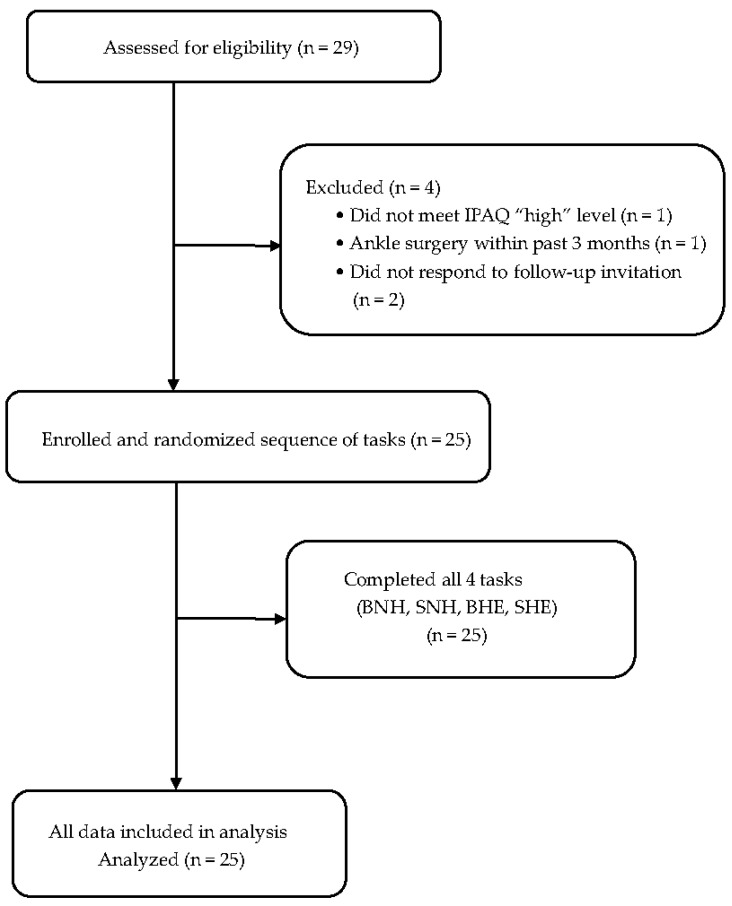
Recruitment and participant flow.

Sample size was determined using G*Power 3.1.9.7 software (Heinrich Heine University Düsseldorf, Germany), with parameters set for a two-tailed paired-samples *t*-test, significance level α = 0.05, effect size *dz* ≈ 0.85, and statistical power = 0.80, yielding a required sample size of 13. Ultimately, 25 healthy male participants (age: 24.52 ± 3.82 years) were recruited between November 2023 and June 2024. Physical activity levels were screened using the International Physical Activity Questionnaire, and only those who scored “high” were included. Baseline anthropometric and physical characteristics of the participants are summarised in Table 1, confirming the homogeneity of the sample. Exclusion criteria comprised neurological, cardiovascular, or metabolic disorders, including lower limb injuries or surgeries within the previous 24 months. Participant recruitment and retention are summarised in the CONSORT-style flow diagram (Figure 1). All procedures were conducted at the Human Performance Laboratory of Waseda University under the supervision of experienced researchers in biomechanics and sports medicine. This study was approved by the Waseda University Ethics Committee (approval number: 2023-314), and all participants provided informed consent according to the Declaration of Helsinki.

### 2.3. EMG Data Collection

**Figure 2:**
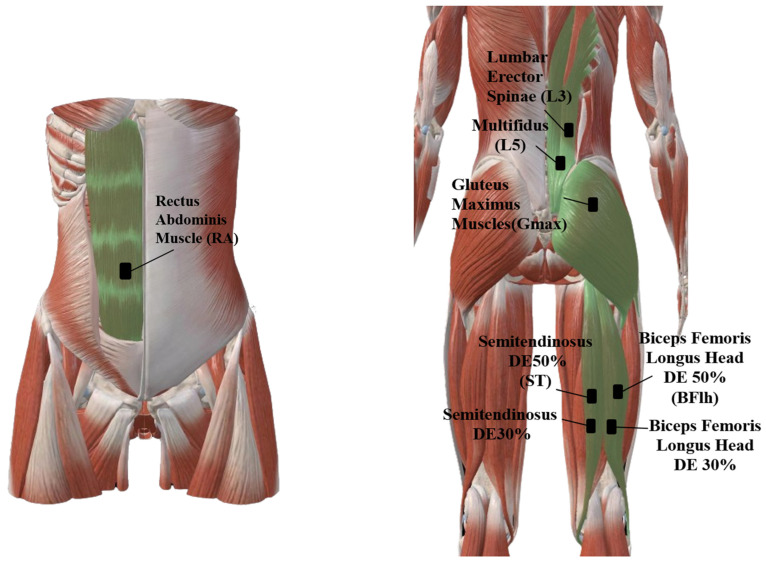
Surface electromyography (EMG) sensor placement locations.

This study employed differential sEMG (Delsys, Inc., Boston, MA, USA; inter-electrode distance: 10 mm; amplification factor: 1000; bandwidth: 20–450 Hz; common mode rejection ratio at 60 Hz > 80 dB; input impedance > 10^15^ Ω/0.2 pF; sampling rate: 2000 Hz). Electrodes were placed on the dominant side (determined as the leg used to kick a ball the furthest) over the rectus abdominis (RA, 4 cm lateral to the umbilicus), the erector spinae at the level of the third lumbar vertebra (L3ES, 4 cm lateral to the L3 spinous process), the multifidus at the level of the fifth lumbar vertebra (L5MF, 2 cm paraspinal to the L5 spinous process), and the gluteus maximus (Gmax, 34% of the distance from the second sacral vertebra to the greater trochanter of the femur). Additionally, electrode sensors were applied to the 50% (BF50, ST50) and distal 30% (BF30, ST30) regions of the long head of the biceps femoris (BF50: midpoint of the line from the ischial tuberosity to the lateral epicondyle of the femur; BF30: 30% of the distal line segment from the ischial tuberosity to the lateral epicondyle) and semitendinosus muscles (ST50: midpoint of the line from the ischial tuberosity to the medial epicondyle of the femur; ST30: 30% of the distal line segment from the ischial tuberosity to the medial epicondyle), also on the dominant side. Specific electrode placement details are provided in Figure 2. EMG measurements were performed on the participant’s dominant leg, consistent with other studies comparing unilateral and bilateral lower limb neuromuscular activation [14].

All electrode sensors were affixed to the skin using double-sided adhesive and aligned approximately parallel to the muscle fibre direction, following SENIAM guidelines [15]. Before electrode placement, the skin was shaved and cleaned with ethanol to enhance adhesion and reduce skin impedance. Electrode positioning was confirmed using ultrasound imaging and isometric contraction against manual resistance.

### 2.4. Kinematic Analysis

A high-speed motion capture system (EX-F100, Casio Computer Co., Ltd., Tokyo, Japan) combined with passive reflective markers (diameter: 15.9 mm) was used to construct a lower limb biomechanical model following the recommendations of the International Society of Biomechanics [16]. The camera was set to record at 120 frames per second and positioned approximately 0.6 m in height and 1.5 m away from the dominant side of the participant. Two-dimensional motion analysis was conducted using Frame-DIAS V software (DKH Inc., Tokyo, Japan). Knee joint flexion angles were calculated by digitising reflective markers placed on the iliac crest, greater trochanter, lateral femoral condyle, and lateral malleolus. The hip joint angle was defined as the angle formed by the line connecting the iliac crest and the greater trochanter and the line connecting the greater trochanter and the lateral femoral condyle. The knee joint angle was defined as the angle formed by the line connecting the greater trochanter and the lateral femoral condyle and the line connecting the lateral femoral condyle and the lateral malleolus. To synchronise kinematic data with EMG recordings, an optical signal from a signal generator (DKH PH-145 all-surrounding optical presenter) was triggered simultaneously with the initiation of EMG recording. This optical signal was captured in real time by the high-speed camera to ensure precise synchronisation.

### 2.5. Testing Procedure

Participants first completed a standardised warm-up consisting of 10 min of jogging (15 laps), followed by three sets of static hamstring stretches (20 s × 3 repetitions). They then practised each of the four interventions—bilateral hip extension (BHE), unilateral hip extension (UHE), bilateral Nordic hamstring curl (BNH), and unilateral Nordic hamstring curl (UNH)—twice under researcher supervision to ensure correct technique and familiarisation, with verbal feedback provided as needed.

BHE was performed in a Roman chair adjusted to the participant’s leg length, with toes planted on the pedals, thighs at ~40° to the floor, and the body held in a straight line (~180°). Participants slowly lowered the upper body through hip and thigh extension, keeping the back upright and arms crossed over the chest, until reaching the maximal achievable angle. UHE followed the same setup, but with one leg fixed and the other suspended; the working thigh remained at ~40° and the upper body straight (~180°). Participants slowly lowered and raised the body through hip flexion of the working leg while maintaining trunk stability.

BNH was performed on a 40° upward-inclined board, with knees flexed, upper body perpendicular to the floor, and feet secured by the researcher. Participants slowly extended the knees from flexion to near full extension via active hamstring contraction, keeping the body straight from head to knees, and leaned forward “as slowly as possible” until control was lost. UNH used the same board, with one leg working and the other suspended. The working leg was secured at the ankle, upper body perpendicular, elbows bent, hands behind the head, and the knee slowly extended to near full extension while maintaining a straight line from head to knee, leaning forward until control was lost.

**Figure 3:**
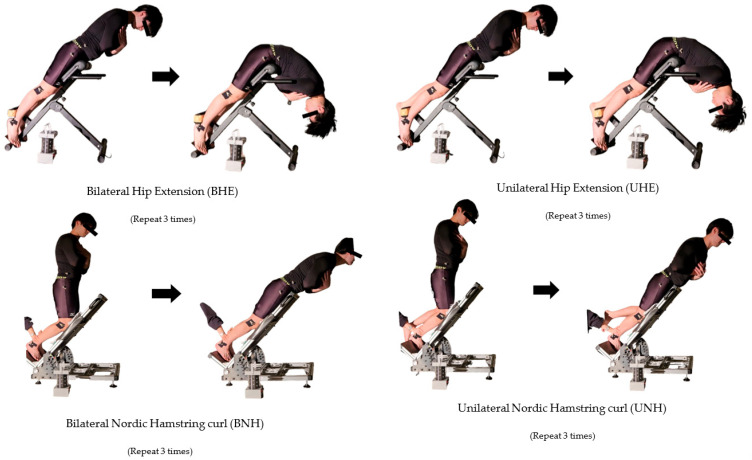
Eccentric training exercises used in the study: BHE, UHE, BNH, and UNH.

Given that the participants in this study were healthy adult males, the existing literature has confirmed that in this population, the maximum unilateral strength and neuromuscular function of the dominant and non-dominant legs exhibit high symmetry without injury, with negligible differences [17]. Therefore, measuring the dominant leg alone is sufficient to represent the typical response of the lower limbs to different movement pattern stimuli. Refer to Figure 3 for visual illustrations of the four exercise interventions.

Following skin preparation (shaving, cleansing with alcohol, and drying), electrodes were applied as described in Section 2.3. Maximal voluntary isometric contractions (MVIC) were then assessed for each target muscle. Two 5 s trials were performed per muscle, with a 1 min rest between trials. The higher of the two values was used for analysis. MVIC testing positions were standardised: prone hip extension for the gluteus maximus, prone knee flexion at 30° and 90° for the hamstrings, prone trunk extension for lumbar extensors, and supine trunk flexion for the rectus abdominis. A metronome set at 60 bpm was used to standardise movement tempo during all interventions. Participants completed three consecutive repetitions of each intervention, with a 1 min rest between repetitions and a 3 min rest between interventions, which were performed in a randomised sequence. Trials were repeated if a movement was not properly executed. If a participant failed >5 times throughout the protocol, their data were excluded to ensure both data reliability and participant safety.

### 2.6. Signal Processing and Feature Extraction

The onset of the eccentric phase was identified using a kinematic-assisted approach. Hip and knee joint angle data, captured via the motion capture system, were smoothed using a 4th-order Butterworth low-pass filter with a cutoff frequency of 6 Hz. Joint velocities and accelerations were then computed using the central difference method [14]. For BHE and UHE, the eccentric phase started when hip angular velocity transitioned from positive (flexion) to negative (extension) [7]. For BNH and UNH, eccentric onset corresponded to knee velocity zero-crossing from positive (flexion) to negative (extension). The biomechanical validity of these points was verified by analysing the delay between these time points and sEMG activation onset (defined as exceeding the baseline mean plus three times the standard deviation [3SD]) for gluteus maximus and biceps femoris, with a strong correlation confirmed (r ≥ 0.68, *p* < 0.05) [20,21]. This kinematic-based approach was adopted because joint velocity zero-crossing provides a mechanically meaningful and reproducible marker of eccentric onset, which has been shown to correspond closely with EMG-defined activation and to minimise variability caused by baseline noise or interindividual differences [22,23]

Raw sEMG data were sampled at 2000 Hz and pre-processed using Delsys EMGworks 4 Analysis (v4.7.4). Signals were first mean-removed to eliminate direct current bias, followed by a 20–450 Hz band-pass filter (4th-order Butterworth). The root mean square (RMS) amplitude was computed using a 100 ms window with 50% overlap, which was used to balance signal stability and temporal resolution, avoiding noise sensitivity of shorter windows and over-smoothing of longer windows [24].

To assess regional recruitment strategies, the RMS ratio of the biceps femoris to semitendinosus (BF/ST) was calculated at both the mid and distal muscle regions. A ratio above 1 indicated BF dominance, whereas a ratio below 1 indicated ST dominance. Proximal-to-distal recruitment was assessed using the RMS50/RMS30 ratio (>1 indicated greater mid-region activation). Additionally, median frequency (MF), torque index (TI), and waveform length (WAV) were extracted using Python (v3.13.0) to evaluate frequency characteristics and fatigue. TI was calculated from the instantaneous RMS of the raw EMG signal weighted by the estimated joint torque:

$$TI=\frac{1}{N}\sum_{i=1}^{N}\frac{RMS(t_{i})}{T(t_{i})}$$

where T($t_{i}$) represents the joint torque at the time sample $t_{i}$, derived from kinematic data obtained via the high-speed motion capture system synchronised with EMG recordings. The detailed kinematic acquisition and processing procedures are described in Section 2.4. Raw RMS values were used in the TI calculation.

WAV was calculated as the cumulative sum of absolute differences between consecutive EMG samples:

$$WAV=\sum_{n=1}^{N-1}\left|x_{n+1}-x_{n}\right|$$

where $x_{n}$ represents the EMG amplitude at sample nnn and N is the total number of samples within the analysed phase.

RMS and WAV values were normalised to the highest MVIC value per muscle rather than the mean. MVICs were performed in triplicate to ensure inter-trial consistency, yielding good reliability (ICC = 0.82).

### 2.7. Data Analysis

All statistical analyses were conducted in Python (v3.13.0) using the Pandas library (v1.3.5). All participant data were analysed using coded labels to ensure blinding of the statistician. Muscles were categorised into core stabilisers (RA, L3ES, and L5MF) and lower limb dynamic muscles (BF50, BF30, ST50, ST30, and Gmax). A two-level comparison was performed: intra-mode (comparing muscles within the same movement) and inter-mode (bilateral vs. single-leg interventions). Normality of the data was assessed using the Shapiro–Wilk test (α = 0.05) and homogeneity of variances using Levene’s test (α = 0.05). Paired-sample *t*-tests were applied, with effect sizes quantified using Cohen’s *dz*. For non-normally distributed data, the Wilcoxon signed-rank test was used, and the rank-biserial correlation (*r*) was calculated as a non-parametric effect size measure. To control for the risk of Type I error due to multiple comparisons, false discovery rate (FDR) correction was additionally performed using the Benjamini–Hochberg procedure. Statistical significance was set at *p_FDR_* < 0.05 after correction.

## 3. Results

### 3.1. Variations in Hamstring Regions and Preferences

**Figure 4:**
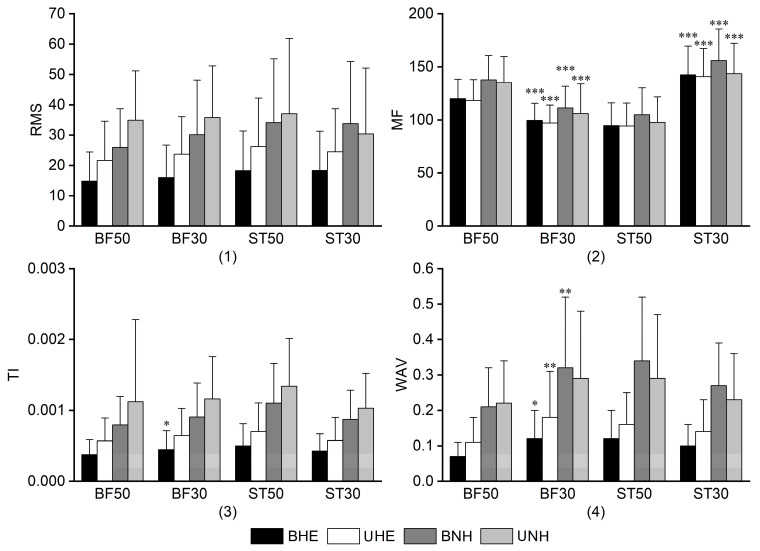
Hamstring region results (**1**) RMS Results; (**2**) MF Results; (**3**) TI Results; (**4**) WAV Results. Note: *, **, and *** indicate significant differences between BHE and UHE or BNH and UNH at *p_FDR_* < 0.05, 0.01, and 0.001, respectively. BF, biceps femoris; BHE, bilateral hip extension; BNH, bilateral Nordic hamstring curl; MF, median frequency; RMS, root mean square; UHE, unilateral hip extension; UNH, unilateral Nordic hamstring curl; ST, semitendinosus; TI, torque index; WAV, waveform length.

As illustrated in Figure 4, no significant differences in RMS values were detected between the mid and distal regions of the hamstrings during identical movement patterns under the same intervention conditions. Regarding MF, consistent patterns were observed across all four intervention modes. BF50 exhibited higher MF values than BF30, with location-based differences ranging from 20% to 28% (all *p_FDR_* < 0.001). The corresponding Cohen’s *dz* was 1.47, 1.37, 1.44, and 1.10, indicating large effects. In contrast, ST50 demonstrated lower MF values than ST30, with a larger discrepancy of 30% to 33%. The paired *t*-test applied to BHE versus UHE revealed significant differences between the two comparisons (df = 24; *dz* = –2.84, *p_FDR_* = 4.41 × 10^−12^; *dz* = –3.24, *p_FDR_* = 2.42 × 10^−13^). Wilcoxon signed-rank tests for the remaining two comparisons revealed large effect sizes (*r* = –0.83, *p_FDR_* = 8.94 × 10^−6^; *r* = –0.84, *p_FDR_* = 5.01 × 10^−6^). Significant differences in TI were detected only under BHE conditions, specifically in the BF muscle, where BF30 exceeded BF50 by over 31% (*r* = −0.48, *p_FDR_* = 0.003). The absolute *r* value was 0.25, suggesting a small effect size. For WAV, BF30 exhibited higher values across all modes except UNH, with the difference between BF50 and BF30 following the order BHE < UHE < BNH (all *p_FDR_* < 0.05). The corresponding absolute *r* was −0.47, −0.57, and −0.65, respectively, indicating small to medium effect sizes. No significant differences were observed in ST.

Table 2 presents the hamstring ratio results. For ratios within the same anatomical site, BF50/ST50 was consistently below one across all intervention modes, whereas BF30/ST30 exceeded one only in UNH, with the remaining results below one. For ratios between different locations of the same muscle, BF50/BF30 remained below one across all interventions, whereas ST50/ST30 exceeded one in all conditions except for BHE, where it was slightly below one. Values close to one indicate similar activation between the compared muscles or regions, reflecting no clear dominance [25]. Statistical comparisons were performed on the underlying RMS values rather than the ratios themselves, ensuring that the reported patterns are supported by significance testing.

### 3.2. Gluteal–Hamstring Complex

#### 3.2.1. RMS

**Figure 5:**
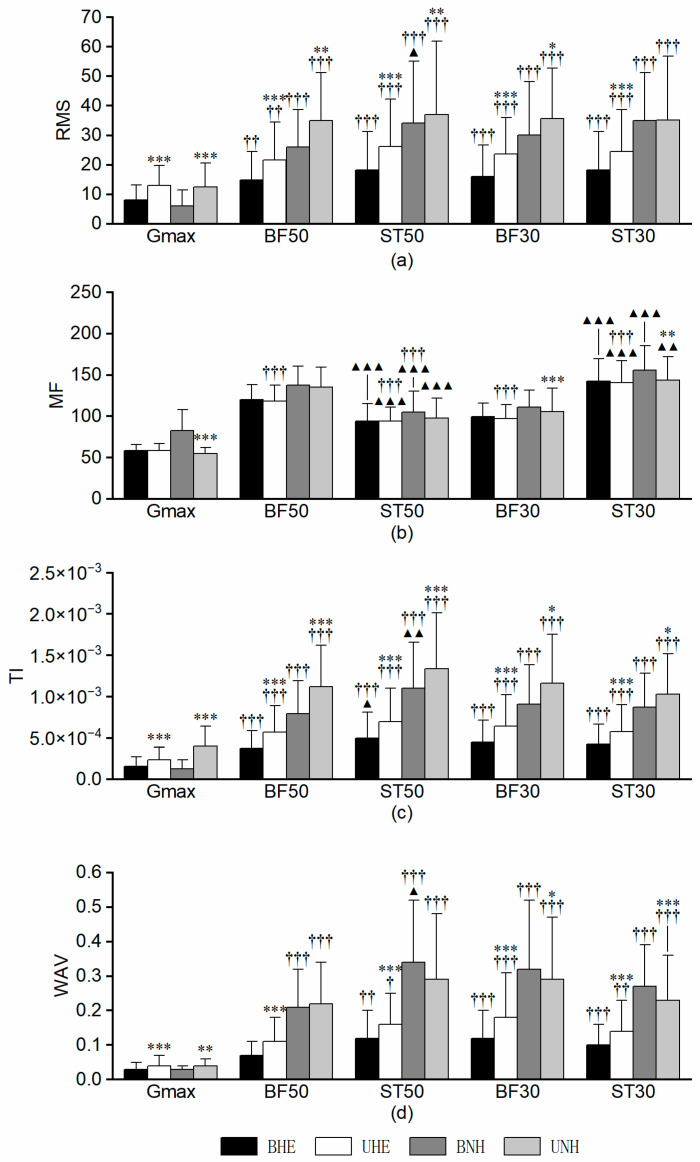
Gluteal–hamstring complex results. (**a**) RMS Results; (**b**) MF Results; (**c**) TI Results; (**d**) WAV Results. Note: *, **, and *** indicate significant differences between BHE and UHE or BNH and UNH (*p_FDR_* < 0.05, 0.01, 0.001). †, ††, and ††† indicate significant differences between Gmax and BF50, ST50, BF30, or ST30 (*p_FDR_* < 0.05, 0.01, 0.001). ▲, ▲▲, and ▲▲▲ indicate significant differences between BF50 and ST50 or BF30 and ST30 (*p_FDR_* < 0.05, 0.01, 0.001). BF, biceps femoris; BHE, bilateral hip extension; BNH, bilateral Nordic hamstring curl; Gmax, gluteus maximus; MF, median frequency; RMS, root mean square; UHE, unilateral hip extension; UNH, unilateral Nordic hamstring curl; ST, semitendinosus; TI, torque index; WAV, waveform length.

As shown in Figure 5a, BF and ST exhibited significantly higher RMS values than Gmax across all four interventions (*dz* = 0.82 to 1.48; *r* = −0.86 to −0.62, all *p_FDR_* < 0.001). Notably, during BNH, ST50 exceeded BF50 by 18.62% (*dz* = −0.48, *p_FDR_* = 0.03), representing the only significant intra-hamstring difference at the same anatomical site.

In the comparison between unilateral and bilateral exercises, both Gmax and BF showed higher RMS values in the unilateral modes (HE and INHE) (all *p_FDR_* < 0.001), with Gmax displaying a more pronounced difference of 51.00% in INHE (df = 24, *dz* = −1.51, *p_FDR_* = 1.99 × 10^−7^). BF30 showed the larger difference of 32.60% in EHE (*r* = −0.85, *p_FDR_* = 2.58 × 10^−6^ ). BF50 also reported higher results in UHE, with a difference of 38.38%. (*r* = −0.29, *p_FDR_* = 4.77 × 10^−6^). For ST50 and ST30, significant differences were exclusively observed in UHE (all *p_FDR_ <* 0.001), with ST50 exceeding BHE by 30.65% (*r* = −0.77, *p_FDR_* = 7.24 × 10^−5^) and ST30 exceeding by 25.41% (*r* = −0.87, *p_FDR_* = 3.58 × 10^−7^).

#### 3.2.2. MF

As shown in Figure 5b, MF displayed highly significant differences across all comparisons (all *p_FDR_* < 0.001). Both BF and ST had higher MF values than Gmax (df = 24, *dz* = 0.74 to 3.46, all *p_FDR_* < 0.001). For intra-hamstring comparisons, the BF50 and ST30 exhibited higher MF values at their respective locations (df = 24, *dz* =−1.90 to 1.34, all *p_FDR_* < 0.001).

In the unilateral–bilateral comparison, ST50, ST30, and Gmax reported higher MF in BNH compared to UNH, with differences of 7.33% (df = 24, *dz* = 0.98, *p_FDR_* = 4.85 × 10^−4^) for ST50, 8.40% (df = 24, *dz* = *0*.77, *p_FDR_* = 0.04) for ST30, and 51.12% (df = 24, *dz* = 1.10, *p_FDR_* = 1.57 × 10^−5^) for Gmax.

#### 3.2.3. TI

As illustrated in Figure 5c, similarly to other parameters, hamstrings demonstrated significantly higher TI values than Gmax across all interventions (*dz* = 0.84 to 2.09; *r* = −0.87 to −0.84, all *p_FDR_* < 0.001). Differences within the hamstrings were only observed in the mid-segment comparison and appeared in only two bilateral patterns. The TI values for BF50 were consistently lower than those for ST50, with differences of 25.00% (df = 24, *dz* = −0.52, *p_FDR_* =.02) and 27.91% (df = 24, *dz* = −0.61, *p_FDR_* = 0.01) under BHE and BNH, respectively. The corresponding Cohen’s *dz* was −0.52 and −0.61, indicating a medium effect.

Across all five sampling sites, unilateral modes demonstrated higher TI. Except for Gmax under INHE conditions, Wilcoxon signed-rank tests were used, reporting large effect sizes (*r* = −0.87 to −0.48, all *p_FDR_* < 0.05). Gmax showed the most pronounced difference in UNH, exceeding BNH by 68.41% (df = 24, *dz* = −1.68, *p_FDR_* = 9.92 × 10^−8^), while all four hamstring sites showed larger TI values in EHE.

#### 3.2.4. WAV

As shown in Figure 5d, the WAV values for ST and BF30 consistently exceeded Gmax (*r* = −0.87 to −0.54, all *p_FDR_* < 0.05). BF50 did not report significance for Gmax in EHE, while it reported a large effect in INHE (*r*_BNH_ = −0.86, *p_FDR_* = 1.25 × 10^−6^; *r*_UNH_ = −0.84, *p_FDR_* = 4.38 × 10^−6^). Furthermore, among all four interventions, only BNH reported significant differences in BF50 and ST50, with a difference of 33.41% (*r* = −0.48, *p_FDR_* = 0.02).

In the unilateral–bilateral comparison, ST50 and ST30 exhibited higher WAV values in BNH (*r*_ST50_ = −0.54, *p_FDR_* = 0.01; *r*_ST30_ = −0.67, *p_FDR_* = 9.30 × 10^−4^), with differences of 95.90% and 66.79%. Meanwhile, they exhibited higher WAV under UNH, though the difference was less pronounced than under EHE (*r*_ST50_ = −0.70, *p_FDR_* =7.01 × 10^−4^; *r*_ST30_ = −0.70, *p_FDR_* = 8.04 × 10^−4^), with respective differences being 38.06% and 51.95%. For Gmax, a significant increase was observed in UHE and UNH (*r*_UHE_ = −0.82, *p_FDR_* = 1.20 × 10^−5^; *r*_UNH_ = −0.64, *p_FDR_* = 0.002), with differences of 50.68% and 5.06%. Differences in BF were only confined to EHE, with 32.20% and 39.71% difference (*r*_BF50_ = −0.77, *p_FDR_* = 2.97 × 10^−5^; *r*_BF30_ = −0.75, *p_FDR_* = 7.69 × 10^−4^), though less significant than in other comparisons (*p* < 0.001).

### 3.3. Trunk Stabiliser Muscles

#### 3.3.1. RMS

**Figure 6:**
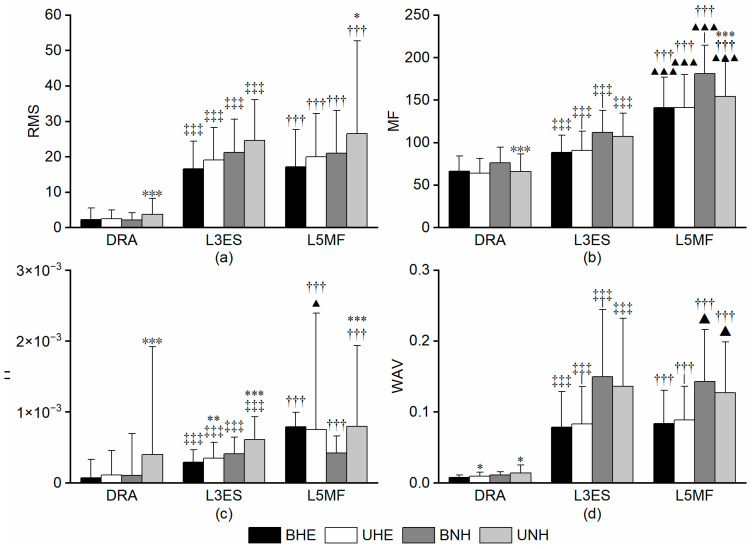
Trunk stabiliser muscle results. (**a**) RMS Results; (**b**) MF Results; (**c**) TI Results; (**d**) WAV Results. Note: *, **, and *** indicate significant differences between BHE and UHE or BNH and UNH (*p_FDR_* < 0.05, 0.01, 0.001). ‡, ‡‡, and ‡‡‡ indicate significant differences between RA and L3ES (*p_FDR_* < 0.05, 0.01, 0.001). †, ††, and ††† indicate significant differences between RA and L5MF (*p_FDR_* < 0.05, 0.01, 0.001). ▲, ▲▲, and ▲▲▲ indicate significant differences between L3ES and L5MF (*p_FDR_* < 0.05, 0.01, 0.001). BHE, bilateral hip extension; BNH, bilateral Nordic hamstring curl; L3ES, erector spinae at the level of the third lumbar vertebra; L5MF, multifidus at the level of the fifth lumbar vertebra; MF, median frequency; RMS, root mean square; UHE, unilateral hip extension; UNH, unilateral Nordic hamstring curl; TI, torque index; WAV, waveform length.

As shown in Figure 6a, both back muscles (L3ES and L5MF) reported significantly higher RMS values than the RA, with differences exceeding 85% (*dz* = −2.16 to −1.60; *r* = −0.85 to −0.88, all *p_FDR_* < 0.001). No significant RMS differences were found between L3ES and L5MF.

In the unilateral–bilateral comparison, RA and L5MF under INHE exhibited significant differences; RA under UNH was 42.16% higher (*r* = −0.71, *p_FDR_* = 3.01 × 10^−4^); L5MF under UNH was 14.58% higher (df = 24, *dz* = −0.48, *p_FDR_* = 0.042).

#### 3.3.2. MF

As illustrated in Figure 6b, MF consistently followed the trend of RA < L3ES < L5MF across all interventions (*dz* = −2.93 to −1.01; *r* = −0.85 to −0.81, all *p_FDR_* < 0.001). The differences between RA and L3ES were at least 25.11%, between RA and L5MF at least 53.03%, and between L3ES and L5MF at least 30.45%.

In the unilateral–bilateral comparison, both RA and L5MF reported higher MF values in BNH compared to UNH, with differences of 15.50% (df = 23, *dz* = 0.96, *p_FDR_* = 1.86 × 10^−4^) and 17.35% (*r* = −0.82, *p_FDR_* = 1.03 × 10^−5^), respectively.

#### 3.3.3. TI

As shown in Figure 6c, a trend of RA < L3ES < L5MF was only evident in UHE, though differences between L3ES and L5MF were less pronounced (*p_FDR_* = 0.04), with corresponding Cliff’s *r* = −0.30, indicating a small effect. In the residual intervention, both L3ES and L5MF significantly outperformed RA (*r* = −0.76 to −0.52, all *p_FDR_* < 0.001).

The TI values demonstrated numerous differences in the unilateral–bilateral comparison. L3ES reported higher TI values in UHE and UNH, though differences in EHE were less significant than in INHE (FDR: *p*_EHE_ = 1.83 × 10^−4^, *p*_INHE_ = 7.15 × 10^−7^), with differences of 37.5% (*r*_EHE_ = −0.66) and 73.57% (*r*_INHE_ = −0.87). For RA and L5MF, significant differences were detected only in INHE, where UNH exceeded BNH (FDR: *p*_INHE_ < 0.001) by 32.79% and 46.54%. The corresponding *r* was −0.87 and −0.74, indicating medium effect sizes.

#### 3.3.4. WAV

As shown in Figure 6d, WAV values followed the pattern RA < L3ES < L5MF. RA exhibited significantly lower values compared to both L3ES and L5MF, with rank-biserial correlations ranging from *r* = −1.0 to 0.63 and all comparisons remaining significant after FDR correction (*p_FDR_* < 0.05). In EHE, only the dorsal muscles (L3ES and L5MF) showed a clear advantage over RA, with r = −1.0 for these comparisons (*p_FDR_* < 0.001), indicating strong and consistent effects despite large absolute differences in mean values.

Only in UHE was a greater difference in DRA observed between unilateral and bilateral movements (df = 23, *dz* = −0.59, *p_FDR_* = 0.01).

## 4. Discussion

This study used sEMG to compare unilateral and bilateral execution of the same eccentric tasks (INHE, EHE), avoiding variability from different movement patterns. Unilateral execution elicited higher peak amplitude and RMS across multiple hamstring regions, reflecting greater neuromuscular demand, whereas frequency–domain parameters, such as MF, were less consistent, suggesting distinct neural control strategies beyond motor unit recruitment. These results provide mechanistic insight into task- and region-specific activation under unilateral loading. As the outcomes reflect acute sEMG features rather than injury endpoints, implications for injury prevention remain hypothesis-generating. Supporting evidence highlights region-specific adaptations and task-dependent neuromuscular strategies [21,26,27,28]. Moreover, EMG responsiveness is intervention-specific; passive self-myofascial release induced only minor, non-significant changes in longissimus dorsi EMG, whereas active unilateral eccentric tasks produced clear amplitude increases, emphasising that task type (passive vs. active eccentric loading) critically shapes acute neuromuscular activation [29].

### 4.1. Neuromuscular Differences Between Unilateral and Bilateral Loading

Regarding the first research question, the results confirm that unilateral loading tends to generate higher neuromuscular activation than bilateral loading within the same eccentric task. This increase in activation may reflect a redistribution of force within the limb, enhanced cortical drive, and greater recruitment of motor units. Mechanistically, unilateral eccentric tasks are thought to engage a larger proportion of high-threshold motor units, thereby promoting greater involvement of fast-twitch fibres—an effect particularly relevant in athletic populations.

This finding aligns with recent work showing that unilateral NHE on a sloped platform elicits significantly higher % MVIC in the BFlh and ST than bilateral executions, particularly at 40–50° incline angles [30]. The added complexity of unilateral tasks, which lack contralateral support, increases demand for pelvic and trunk stability. This observation is consistent with prior findings in exercises like squats and lunges [28]. A reduced base of support also necessitates more precise limb control. Among sEMG parameters, amplitude-based metrics (e.g., RMS, peak amplitude) were more sensitive to differences in limb symmetry, whereas frequency-based variables (MF, TI, WAV) exhibited less consistent changes, suggesting that recruitment intensity rather than discharge rate modulation primarily drives the observed differences [21,26].

### 4.2. Regional Functional Specialisation of the Hamstrings

The study also revealed consistent regional differences in neuromuscular parameters, particularly in MF, TI, and variability of motor unit recruitment (WAV). Consistent with findings by Smith et al. [31], the mid-belly and distal regions of the hamstrings were activated concurrently, and the quality and timing of motor unit recruitment differed between regions. This suggests a complex pattern of intramuscular coordination. For instance, the distal BFlh exhibited higher MF values, indicative of faster and more fatigue-prone motor units, whereas the mid-belly of the ST showed higher TI and WAV, suggesting greater torque contribution and more diverse recruitment patterns.

These results are consistent with previous reports highlighting region-dependent activation patterns in eccentric hamstring tasks [27,28]. Recent longitudinal work also shows that distal regions of BFlh demonstrate more pronounced morphological adaptations after Nordic hamstring training (e.g., increased sarcomere number and muscle volume) compared to central regions, reinforcing the importance of accounting for spatial heterogeneity when designing targeted eccentric programs [27]. Furthermore, a complementary recruitment strategy was observed between the fascicle-dominant BFlh and the tendon-dominant ST; mid-BFlh showed reduced activation, while ST mid-belly increased, aligning with respective torque demands. Notably, unilateral INHE elicited an increased BF30/ST30 activation ratio, suggesting an adaptive compensation mechanism that distributes mechanical load and may protect tendon insertion sites.

### 4.3. Practical Applications

The neuromuscular differences between unilateral and bilateral EHE highlight practical implications for performance and rehabilitation. Unilateral execution produced greater, region-specific activation, suggesting potential benefits for power development and joint stability in tasks requiring rapid deceleration, directional changes, or asymmetrical control. While these findings reflect acute demand and require longitudinal confirmation, they support prioritising unilateral eccentric work to enhance neural drive and address asymmetries common in sprinting, cutting, and striking sports [29].

In rehabilitation contexts, unilateral eccentric modalities may provide a controlled environment to retrain neuromuscular coordination in a limb-specific manner, particularly useful when bilateral loading is contraindicated due to pain, injury, or asymmetry. The enhanced proprioceptive demand of unilateral tasks can also facilitate reactivation of stabilising musculature, supporting neuromuscular re-education. Recent intervention studies suggest that unilateral flywheel hip extension and leg curl training significantly increase eccentric peak power, although concentric and isometric strengths may not change substantially [28]. This evidence highlights the potential of unilateral eccentric modalities for injury prevention, rehabilitation, and individualised training prescription.

### 4.4. Limitations and Future Directions

Several limitations should be acknowledged. First, although sEMG was employed across multiple muscles and parameters, the absence of concurrent kinetic and kinematic data limits direct interpretation of the mechanical underpinnings of the observed differences. Second, only healthy young male participants were included, restricting generalisability to female athletes, older adults, or clinical populations (e.g., ACL-injured individuals). Third, longitudinal intervention studies are warranted to determine whether the observed acute activation differences translate into clinically meaningful outcomes, such as strength gains, performance enhancement, or injury reduction. Finally, the study did not track injury incidence or re-injury; therefore, the findings should not be interpreted as evidence of injury risk reduction. Future work should integrate prospective surveillance or randomised interventions to link acute activation profiles with injury outcomes.

Despite these limitations, the present study offers several novel contributions. It is the first to systematically compare unilateral and bilateral eccentric movements using a multidimensional sEMG framework encompassing amplitude-, timing-, and fatigue-related features while controlling for exercise type. By isolating limb configuration effects, these findings enhance our understanding of motor strategies and provide guidance for individualised eccentric training programs. Integrating EMG markers with longitudinal monitoring could further inform injury risk mitigation and performance optimisation [21,26,27,28]. To strengthen reproducibility, this study also clarifies SENIAM-consistent electrode placement and, for variables violating normality, reports the median [IQR] with non-parametric corroboration alongside parametric tests, an approach consistent with recent EMG research [29].

## 5. Conclusions

This study systematically examined neuromuscular activation differences between unilateral and bilateral executions of the same eccentric training tasks—INHE and EHE—using a multidimensional sEMG framework. By isolating the effect of movement symmetry within matched exercise types, this study avoided the confounding influence of comparing fundamentally different movement patterns. These findings confirmed that unilateral loading generally elicited greater neuromuscular activation, particularly in amplitude-based parameters, while regional differences in frequency–domain features reflected distinct motor control strategies. These results directly address the research questions, demonstrating that exercise symmetry and anatomical muscle specialisation jointly shape neuromuscular recruitment patterns.

These findings have practical implications for designing eccentric training programs to enhance performance and correct side-to-side imbalances. While their relevance to injury risk remains provisional, requiring prospective studies linking acute activation patterns to injury outcomes, the observed compensatory interplay between semitendinosus and biceps femoris segments indicates that targeted unilateral exercises can optimise regional muscle activation, potentially informing tendon-specific rehabilitation strategies.

Future research should explore whether these acute activation differences translate into long-term functional improvements, such as strength gains, sprint performance, or injury risk reduction. Studies including female athletes, older adults, and clinical populations are needed to verify the generalisability of these findings. Additionally, integrating kinematic, kinetic, or imaging-based assessments could provide deeper mechanistic insights into the observed neuromuscular patterns. Overall, this study advances our understanding of how symmetry and regional specialisation influence eccentric muscle function, providing a foundation for evidence-based training and rehabilitation interventions.

## Figures and Tables

**Table 1:** Baseline characteristics of participants.

	Mean ± Standard Deviation
**Age (years)**	24.52 ± 3.74
**Height (cm)**	175.53 ± 5.33
**Weight (kg)**	72.06 ± 7.29
**BMI**	23.13 ± 2.57

BMI, body mass index; IPAQ.

**Table 2:** Inter-electrode sEMG ratios under different exercise modes.

	BHE	UHE	BNH	UNH
**BF50/ST50**	0.81	0.82	0.76	0.94
**BF30/ST30**	0.87	0.97	0.89	1.02
**BF50/BF30**	0.93	0.91	0.86	0.98
**ST50/ST30**	1.00	1.07	1.01	1.05

## Data Availability

The data that support the findings of this study are available from the corresponding author upon reasonable request.

## Supplementary Materials

The following supporting information can be downloaded at https://www.mdpi.com/article/10.3390/sports13100364/s1, Table S1: CONSORT 2025 checklist.

## Abbreviations

The following abbreviations are used in this manuscript:

BF	biceps femoris
BHE	bilateral hip extension
BNH	bilateral Nordic hamstring curl
EHE	eccentric hip extension
EMG	electromyography
Gmax	gluteus maximus
INHE	inclined Nordic hamstring exercise
L3ES	erector spinae at the level of the third lumbar vertebra
L5MF	multifidus at the level of the fifth lumbar vertebra
MF	median frequency
MVIC	maximal voluntary isometric contractions
NHE	Nordic hamstring exercise
UHE	unilateral hip extension
UNH	unilateral Nordic hamstring curl
RA	rectus abdominis
RMS	root mean square
sEMG	surface electromyography
ST	semitendinosus
TI	torque index
WAV	waveform length
